# Artificial intelligence in ophthalmology: trust, bias, and responsibility from the perspective of medical students and ophthalmologists

**DOI:** 10.3389/fopht.2026.1766974

**Published:** 2026-03-03

**Authors:** Yacoub A. Yousef, Areen Shdeifat, Leen Yousef, Mona Mohammad, Tamara AlNawaiseh, Leena Abdullah, Mutasem Elfalah, Iyad Sultan, Ibrahim AlNawaiseh

**Affiliations:** 1 Departments of Surgery (Ophthalmology), King Hussein Cancer Centre (KHCC), Amman, Jordan; 2 Department of Special Surgery, Faculty of Medicine, The University of Jordan, Amman, Jordan; 3 International General Certificate of Secondary Education (IGCSE), Islamic Educational College, Amman, Jordan; 4 Department of Pediatric Oncology, King Hussein Cancer Centre (KHCC), Amman, Jordan

**Keywords:** artificial intelligence, clinical decision-making, medical education, ophthalmology, trust in AI

## Abstract

**Background:**

Artificial intelligence (AI) is increasingly integrated into ophthalmology, offering advances in diagnostic accuracy and surgical decision support. However, perceptions, trust, and ethical concerns regarding AI among medical students and ophthalmologists remain insufficiently explored.

**Methods:**

A cross-sectional survey was conducted among 525 participants, including 353 medical students and 172 ophthalmologists. The questionnaire assessed perceptions of diagnostic reliability, AI-assisted surgical outcomes, responsibility attribution, ethical concerns, and trust in AI compared with clinician judgment.

**Results:**

Most participants in both groups perceived human clinical expertise as more reliable for diagnosis than AI-driven systems (medical students 80%; ophthalmologists 72%; p = 0.054). In contrast, more than half of respondents believed AI-assisted surgery could achieve superior outcomes compared with manual techniques (medical students 55%; ophthalmologists 56%). Primary responsibility for AI-related clinical outcomes was most commonly attributed to physicians rather than AI developers (medical students 62%; ophthalmologists 66%; p = 0.666), and bias was identified as the leading ethical concern (70% of medical students and 75% of ophthalmologists). Approximately 70% of participants viewed AI as a complementary tool rather than a replacement for ophthalmologists, although nearly half anticipated AI might replace some optometric functions. In human–AI disagreement scenarios, trust was context-dependent: 77–79% deferred to AI when it contraindicated surgery recommended by clinicians, whereas 91% favored clinician judgment when AI recommended surgery against clinical advice. Early-career ophthalmologists demonstrated greater support for AI-assisted surgery compared with senior colleagues (p = 0.013).

**Conclusion:**

Both medical students and ophthalmologists recognize AI’s potential in ophthalmology, particularly for surgical applications, while continuing to prioritize human expertise for diagnosis. AI is largely viewed as a complementary tool, with ethical concerns surrounding bias and responsibility remaining prominent. Trust in AI varies by clinical context, and acceptance of AI-assisted surgery is greater among early-career ophthalmologists.

## Introduction

Artificial intelligence (AI) plays an important role in transforming healthcare by enhancing diagnostic accuracy and enabling personalized and precision-based decisions. Ophthalmology, for example, has multiple AI applications in disease diagnosis, guiding treatment plans, surgical advice, and imaging interpretations, especially in retinal diseases such as diabetic retinopathy and age-related macular degeneration, where fundus photos can be read by AI-driven systems (1). In addition to computational power, it supports clinicians’ decision-making, boosts efficiency, and may reduce human errors (2).

However, integration of AI into ophthalmic practice raises many concerns around trust, accountability, and the evolving clinician role. Many previous surveys and reviews showed that eye care professionals are optimistic about these great advancements, but they have significant concerns about AI’s reliability, ethical implications, and risks of professional displacement (3–7).

In this study, we analyzed the perceptions of both medical students and practicing ophthalmologists (the current and future ophthalmologists) toward AI use in ophthalmology that may help in shaping ethical frameworks and educational strategies about the implementation of AI as a potential part of the standard of care in the field of ophthalmology.

## Methods

The institutional review board (IRB) committee at King Hussein Cancer Center, Amman, Jordan, approved this cross-sectional study (25KHCC004).

### Study design and participants

We performed a cross-sectional survey of 525 participants: 353 medical students and 172 ophthalmologists. Participants were recruited using a convenience sampling approach through institutional mailing lists and professional networks affiliated with academic medical centers like the University of Jordan and the Jordanian Ophthalmic Society, with a completion count of n=525. Eligible participants included medical students, ophthalmology residents, and practicing ophthalmologists. Participation was voluntary and anonymous. Medical students enrolled in accredited medical schools and ophthalmologists at any stage of training or practice were eligible to participate. Incomplete survey responses were excluded from the final analysis. The ophthalmologist group was stratified based on years of clinical experience into three groups: Residents in training, practicing ophthalmologists with <10 years of experience, and practicing ophthalmologists with >10 years of experience. As participation was voluntary and based on convenience sampling, selection bias cannot be excluded, and the study sample may overrepresent individuals with greater interest or familiarity with artificial intelligence.

### Survey instrument and validation

The questionnaire was adapted from previously published and validated instruments assessing perceptions of artificial intelligence among eye care professionals and healthcare providers (3). The original tools were modified to ensure relevance to ophthalmology practice by tailoring terminology, selecting context-specific items, and incorporating human–AI disagreement scenarios relevant to diagnostic and surgical decision-making. Demographic data included gender, training level, and years of experience. Internal consistency reliability of the scale was evaluated using Cronbach’s alpha (α) and Guttman’s lambda-2 (λ_2_). Cronbach’s alpha for the scale was 0.64, indicating a questionable low internal consistency under the assumption of tau-equivalence. Because this assumption doesn’t hold for the present data, Guttman’s lambda-2, which is less sensitive to unequal item loadings, was also computed. The lambda-2 coefficient was 0.72, suggesting acceptable reliability. Item-rest correlations showed moderate associations across items, supporting internal coherence of the instrument.

### Survey-assessed parameters

1. Diagnostic reliability: preference of human expertise vs. AI diagnosis capability.
2. Surgical outcomes: perceptions of AI-assisted vs. traditional surgery.
3. Responsibility attribution: physicians vs. AI developers for AI-driven outcomes.
4. Challenges: concerns about bias, ethics, and healthcare disparities.
5. Trust in AI vs. clinicians: responses to hypothetical clinical-AI conflicts.

### Data collection and analysis

This study was exploratory in nature. Given the multiple comparisons performed, no formal adjustment for multiple testing was applied. The analyses were intended to be descriptive and hypothesis-generating rather than confirmatory, and the potential risk of false-positive results is acknowledged. Descriptive statistics and chi-square tests compared categorical data. P value less than 0.05 was considered significant.

## Results

### Participant demographics

Among 525 respondents, 353 (67%) were medical students and 172 (33%) were ophthalmologists. Ophthalmologists included residents (35%), those with <10 years’ practice (38%), and those with>10 years (26%). Most participants were female (67%) (**Table 1**).

**Table 1:** Participants’ Demographics.

Variable	Total	Medical Students	Ophthalmologists
Total number	525	353 (67%)	172 (33%)
Gender			
Female	352 (67%)	272 (77%)	80 (47%)
Male	173 (33%)	81 (23%)	92 (53%)
Age			
Under 48	393 (75%)	353 (100%)	40 (23%)
48–80 Year	97 (18%)	0	97 (57%)
More than 80	35 (7%)	0	35 (20%)
Experience in AI	225 (43%)	166 (74%)	59 (34%)

### Perceptions of AI integration

Most participants favored human expertise for diagnosis, while over half viewed AI-assisted surgery as potentially beneficial. Physicians were seen as primarily responsible for AI outcomes, and algorithmic bias was cited more than healthcare disparities as the main ethical concern. No significant differences were observed between students and ophthalmologists (**Table 2**).

**Table 2:** Perceptions of medical students and ophthalmologists regarding artificial intelligence in ophthalmology practice.

Question	Choices	Medical students (353) (N, %)		Ophthalmologists (172) (N, %)		P value
Reliability	AI-driven systems	72	20%	48	28%	0.054
	Human expertise	281	80%	124	72%	
Outcomes	AI-assisted surgery	193	55%	96	56%	0.851
	Manual surgery	160	45%	76	44%	
Responsibility	Physician	219	62%	113	66%	0.666
	Developers	134	38%	59	33%	
Challenges	Healthcare disparities	107	30%	43	25%	0.206
	Bias and ethics	246	70%	129	75%	
Opportunities (Ophthalmologists)	Complement and create	248	70%	120	70%	0.908
	Replace roles	105	30%	52	30%	
Opportunities (Optometry)	Complement and create	179	51%	80	47%	0.366
	Replace roles	174	49%	92	53%	
Skills	Enhance skills	232	66%	110	64%	0.689
	Reduce skills	121	34%	62	36%	
Relationship	Strengthen relationship	156	44%	76	44%	0.998
	Weaken relationship	197	56%	96	56%	
Expected outcome	AI recommendations	36	10%	11	6%	0.152
	Human expertise	317	90%	161	94%	
Training	Collaborate with AI	277	78%	140	81%	0.436
	Traditional techniques	76	22%	32	19%	
Leadership	Lead in diagnosis	116	33%	57	33%	0.84
	Lead in all	156	44%	80	47%	
	Not lead	81	23%	35	20%	
Choice	Rely on AI	62	18%	11	6%	0.0005
	Supportive tool	291	82%	161	94%	
Role	disruptive tool	17	5%	11	6%	0.354
	Supportive tool	240	70%	107	62%	
	transformative force	96	27%	54	32%	

### Trust in clinical decision-making

In paradoxical scenarios, 90% of students and 94% of ophthalmologists trusted human judgment over AI. Over two-thirds supported AI collaboration in education. Ophthalmologists were more likely than students to see AI as supportive rather than replacing human decisions (94% vs. 82%; **Table 2**).

### Analysis of clinical–AI conflicts

Participants showed asymmetric trust: 77–79% followed AI when it contraindicated surgery, whereas 91% favored clinicians when AI recommended surgery against clinical advice.

### Experience-related trends

Ophthalmologists with <10 years’ experience or in training were more supportive of AI-assisted surgery than those with >10 years (p = 0.013). No other experience-related differences were observed (**Table 3**).

**Table 3:** Subgroup analysis of ophthalmologists’ attitudes toward ai based on experience level.

Question	Choices	Ophthalmologists >10 years (N = 45)		Ophthalmologists <10 years (n=66)		residents (n=61)		P value
Reliability	AI-driven systems	17	38%	17	26%	12	20%	0.051
	Human expertise	28	62%	49	74%	49	80%	
Outcomes	AI-assisted surgery	9	20%	31	47%	28	46%	0.013
	Manual surgery	36	80%	35	53%	33	54%	
Responsibility	Physician	32	71%	46	70%	39	64%	0.605
	Developers	13	29%	20	30%	22	36%	
Challenges	Healthcare disparities	15	33%	12	18%	16	26%	0.133
	Bias and ethics	30	67%	54	82%	45	74%	
Opportunities (Ophthalmologists)	Complement and create	36	80%	41	62%	44	72%	0.099
	Replace roles	9	20%	25	38%	17	28%	
Opportunities (Optometry)	Complement and create	24	53%	26	39%	28	46%	0.226
	Replace roles	21	47%	40	61%	33	54%	
Skills	Enhance skills	35	78%	43	65%	36	59%	0.057
	Reduce skills	10	22%	23	35%	25	41%	
Relationship	Strengthen relationship	21	47%	30	45%	25	41%	0.696
	Weaken relationship	24	53%	36	55%	36	59%	
Expected outcome	Human expertise	42	93%	62	94%	57	93%	0.931
	AI recommendations	3	7%	4	6%	4	7%	
Training	Collaborate with AI	39	87%	56	85%	47	77%	0.498
	Traditional techniques	6	13%	11	15%	14	23%	
Leadership	Lead in diagnosis	14	31%	24	36%	12	20%	0.66
	Lead in all	25	56%	32	49%	22	36%	
	Not lead	6	13%	10	15%	17	28%	
Choice	Rely on AI	3	7%	6	9%	1	2%	0.776
	Supportive tool	42	93%	60	91%	60	98%	
Role	Transformative tool	14	31%	22	33%	17	28%	0.921
	Supportive tool	28	62%	38	58%	43	71%	
	Disruptive force	3	7%	6	9%	1	2%	

## Discussion

The results of our study provide a comprehensive analysis of the perceptions of both medical students and ophthalmologists in a homogenous community towards the use of artificial intelligence (AI) in ophthalmology practice. Overall, our findings showed that AI is preferred as a complementary supportive tool that can help in diagnosis and recommendations for treatment, rather than a replacement for human expertise. This is expected because of the known promising AI capabilities and potential, but with a substantial limitations and ethical challenges (8–10). However, it is important to emphasize that our findings reflect perceptions and attitudes derived from hypothetical scenarios rather than observed clinical behavior.

Diagnostic reliability remains a major concern for practicing physicians when it comes to the integration of AI into clinical practice. Eighty percent of medical students and 72% of ophthalmologists still favor human clinical judgment over AI in diagnostic decision-making, which is consistent with prior literature emphasizing the importance of contextual and experimental knowledge that AI systems often lack (11). Diagnostic process in ophthalmology involves making correlations between clinical findings, patient history, and disease manifestations that current AI models may not fully capture, especially given their training on limited or homogeneous datasets. This underscores the necessity for clinicians to use AI cautiously, rather than deferring blindly. However, the perception that AI-assisted surgical techniques and recommendations could yield a better outcome was endorsed by more than half of participants in this study (55% of medical students and 56% of ophthalmologists), highlighting a growing confidence in AI’s ability to enhance procedural precision and consistency (12). Surgical applications of AI, such as robotic-assisted microsurgery and intraoperative guidance, have demonstrated promise in improving outcomes by minimizing human error, reducing fatigue-related variability, and enabling data-driven customization of surgical plans. This growing confidence in AI capabilities and in machine learning in general comes from the understanding that the AI mechanism of work depends on repetitive, high-precision tasks, and surgeons believe that these capabilities can help and enhance a surgeon’s performance and surgical outcomes. This perspective is consistent with findings from other fields, including neurosurgery and orthopedics (11, 13).

Surprisingly, we find that the trust in AI is variable according to the clinical scenarios involving a paradox between clinical human judgment and AI recommendations. When AI contradicted a clinician’s positive assessment of the expected outcome for refractive surgery, the majority (77%) deferred to AI recommendations, suggesting that participants regard AI as an important safeguard against overtreatment and potential procedural risks. This tendency was even stronger among medical students (79%) compared to ophthalmologists (72%), which indicates that less clinical experience leads to greater reliance on algorithmic caution than on human judgment. In contrast, when AI claimed that refractive surgery is safe and recommended in a situation where clinicians advised against this procedure, 91% of participants sided with the clinicians, reflecting enduring confidence in human expertise to make high-stakes, risk-averse decisions.

This asymmetry in trust distribution highlights a key psychological behavior: AI is predominantly trusted as a risk mitigation tool, especially in identifying when intervention may be unwarranted or harmful, but human clinical judgment retains primacy in approving high-risk procedures (13, 14). Such dynamics have been reported in radiology and oncology, where AI is welcomed for flagging suspicious findings or predicting adverse events but is less trusted to overrule experienced clinicians’ decisions to withhold treatment (11, 13). This phenomenon also highlights the importance of understanding AI not as an autonomous decision-maker but as a collaborator that enhances clinical safety. These interpretations should be considered cautiously, as psychological explanations for trust dynamics were not directly measured and remain speculative.

Ethical and practical concerns about AI’s implementation were prominent. The leading ethical concern in this study was focused on bias and ethics rather than healthcare disparities, as advocated by 69% of medical students and 76% of ophthalmologists. These concerns are well-founded, given that AI algorithms often derive from datasets that lack diversity, leading to potential misdiagnoses or inequitable care in underrepresented populations (15). The risk of systemic biases through AI could exacerbate existing health disparities unless human efforts are made to validate the algorithms to ensure fairness and transparency. Additionally, concerns about accountability because clinicians found it hard to trust AI models whose decision pathways are not clearly understood by humans, raising challenges in clinical validation and medico-legal liability (16, 17).

The vast majority of participants still believe that the responsibility for AI-driven clinical outcomes should be assigned to physicians rather than AI developers, and this aligns with current ethical and regulatory paradigms that place ultimate accountability on human clinicians. This finding emphasizes the importance of continuous clinical validation of all AI tools and for clear regulatory guidelines that define the boundaries of AI’s clinical role. Without such frameworks, there is a risk of legal ambiguity and erosion of trust among both practitioners and patients.

As expected, our data showed certain differences in perception toward AI within the ophthalmologist cohort. Those with fewer than 10 years of experience or who are ophthalmology residents in training were significantly more supportive of AI-assisted surgical interventions than ophthalmologists with more than 10 years of practicing experience. This trend likely reflects increased exposure to digital technologies during training and a greater openness to technological innovation in the young group (18, 19). It also suggests that early and sustained incorporation of AI literacy into medical education could foster more uniform acceptance and more effective human-AI collaboration in future clinical practice. More senior physicians are still trusting human skills more. This explains why about one-third of participants are worried that increased reliance on AI could impair the clinical judgment capabilities and worsen the surgical skills among future physicians (10, 19). The greater enthusiasm for AI-assisted surgery observed among residents and early-career ophthalmologists may reflect several factors beyond digital familiarity alone. These include differences in training environments, levels of surgical autonomy, perceived medico-legal responsibility, risk tolerance, and greater exposure to supervised or technology-augmented surgical workflows during training. In contrast, senior ophthalmologists may rely more heavily on experiential judgment developed over years of independent practice, contributing to more cautious adoption of AI-assisted interventions. Furthermore, more than half of the participants also believed that AI might negatively affect the doctor-patient relationship, possibly out of concern that it could make care feel less personal or reduce the sense of empathy. These findings underscore the importance of integrating AI in ways that uphold the human side of medicine, emphasizing communication, empathy, and ethical responsibility.

The support for incorporating AI training into ophthalmology curricula (69-71%) is encouraging, as it is a new tendency in medical education towards creating a structured, longitudinal education to improved algorithm literacy to ensure clinicians develop the skills necessary to critically appraise and effectively use AI tools (15, 19). Such educational initiatives will be crucial to avoid both over-reliance and undue skepticism and to cultivate the competence and confidence necessary for future ophthalmologists getting the maximum benefits of AI safely.

While our study provides valuable insights, it is important to consider its limitations. The use of hypothetical clinical scenarios, while methodologically necessary to control variables, may not capture the full complexity and time pressures inherent in real-world clinical decision-making. As respondents’ answers reflect idealized rather than practical behaviors, these findings should be viewed as a baseline for future qualitative study. Furthermore, the sample likely includes participants with more interest or familiarity with AI, potentially biasing results toward more favorable views. The study’s regional focus may limit generalizability, as attitudes toward AI can vary widely based on cultural, institutional, and healthcare system differences (20). Future research should explore these factors in a diverse larger population. Additionally, the regional nature of the study population may limit generalizability, as attitudes toward artificial intelligence can vary across healthcare systems, regulatory environments, and cultural contexts.

In summary, AI in ophthalmology is a promising, yet context-dependent, adjunct whose greatest value lies in complementing human clinical judgment rather than replacing it (21). The nuanced interplay of trust, risk perception, and ethical considerations revealed here should guide future AI deployment in clinical practice. We recommend integrating AI education throughout medical training, establishing clear ethical and regulatory frameworks, and designing specialty-specific models of human-AI collaboration that optimize patient outcomes without compromising the clinical role of physicians. This balanced approach will be essential as ophthalmology navigates the transformative potential of AI.

## Conclusion

Artificial intelligence is a valuable tool in ophthalmology, with trust depending mainly on clinical context and perceived risk. AI is most trusted to avoid over-treatment, while clinician judgment remains decisive in high-stakes decisions. To maximize AI benefits, we need to integrate AI training in medical education, clarify accountability, and collaborative frameworks by making ethical guidelines that regulate human-AI interaction. Combining clinical sense and AI algorithms will be key to future ophthalmic practice.

## Data Availability

The raw data supporting the conclusions of this article will be made available by the authors, without undue reservation.

## Supplementary material

SUPPLEMENTARY FILE 1 Summary of survey-assessed parameters and demographics.
