# Supplementary material for: Artificial intelligence in ophthalmology: trust, bias, and responsibility from the perspective of medical students and ophthalmologists
Source: Front Ophthalmol (Lausanne). 2026 Mar 3;6:1766974. doi: 10.3389/fopht.2026.1766974 (PMC12991976; doi:10.3389/fopht.2026.1766974)
Supplement: SUPPLEMENTARY FILE 1 — Summary of survey-assessed parameters and demographics. [file Supplementaryfile1.doc]

Participant’s information:

**Gender:** Male

Female

**Age group**: < 20 yrs. 20-25 yrs. 25-30 yrs. 30-40 yrs.

40-50 yrs. 50-60 yrs. > 60 yrs.

**Level of education**: Medical student,

Ophthalmologist in training,

Ophthalmologist in practice (<10 years)

Ophthalmologist in practice (>10 years)

### Questionnaire on Anticipation of AI Use in Ophthalmology

1. **Which do you think is more reliable for accurate diagnoses and decision-making in ophthalmology?**

AI-driven systems with advanced algorithms

Human expertise based on clinical experience

1. **What approach do you believe will likely result in better surgical outcomes during ophthalmic surgery?**

AI-assisted surgery

Traditional manual surgery

1. **In cases of AI-related errors, who should bear primary responsibility?**

The physician overseeing the process

The developers or manufacturers of the AI system

1. **Which of the following concerns do you think poses a bigger challenge with the adoption of AI in ophthalmology?**

Reducing healthcare disparities

Potential bias, data misuse, and ethical concerns

1. **What impact do you believe AI will have on job opportunities for ophthalmologists?**

AI will complement and create new roles for ophthalmologists

AI will replace many traditional roles in ophthalmology

1. **What impact do you believe AI will have on job opportunities for optometry?**
   1. AI will complement and create new roles for optometry
   2. AI will replace many traditional roles in optometry
2. **How do you think AI will affect the development of surgical skills in future ophthalmologists?**

It will enhance skills by providing advanced tools and simulations

It will reduce hands-on practice, potentially diminishing skill development

1. **How do you think AI will affect the patient-doctor relationship in ophthalmology?**

Strengthen relationships by allowing more time for patient care

Weaken the bond due to reduced direct interactions

1. **In cases of a complete paradox regarding the expected outcome, who should make the final decision?**

Human expertise and clinical judgment

AI’s data-driven recommendations

1. **What type of training should future ophthalmologists receive?**

Focus on collaborating with AI systems for both diagnosis and surgery

Adhere strictly to traditional clinical and surgical techniques

1. **Do you believe AI in ophthalmology should:**

Lead in diagnosis and decision-making but not in surgical recommendations

Lead in diagnosis, decision-making, and surgical recommendations

Not lead in diagnosis, decision-making, or surgical recommendations

1. **If you had to choose one option, which would you select?**

Rely completely on AI decisions in validated systems, ignoring traditional principles in diagnosis and management

Use AI only as a supportive tool and rely primarily on human decision-making, even if AI recommendations contradict it

1. **How do you perceive the overall role of AI in the future of ophthalmology?**

A transformative tool that enhances both diagnosis and treatment

A supportive tool with limitations that require oversight

A disruptive force that may undermine traditional practices

1. **You are the patient: Your ophthalmologist assures you that refractive surgery is safe for you, but an AI-driven algorithm suggests it is not safe. Which decision would you trust?**

Ophthalmologist expertise and clinical judgment

AI’s data-driven recommendations

1. **You are the patient: Your ophthalmologist advises that refractive surgery is not safe for you, but an AI-driven algorithm suggests it is safe. Which decision would you trust?**

Ophthalmologist expertise and clinical judgment

AI’s data-driven recommendations
